# Perceived control over condom use among sex workers in Madagascar: a cohort study

**DOI:** 10.1186/1472-6874-10-4

**Published:** 2010-01-28

**Authors:** Audrey Pettifor, Abigail Norris Turner, Teresa Swezey, Maria Khan, Mbolatiana SM Raharinivo, Bodo Randrianasolo, Ana Penman-Aguilar, Kathleen Van Damme, Denise J Jamieson, Frieda Behets

**Affiliations:** 1 Department of Epidemiology, University of North Carolina, Chapel Hill, USA; 2 UNC-MAD, Antananarivo, Madagascar; 3 Division of Reproductive Health, Centers for Disease Control and Prevention, Atlanta, Georgia, USA

## Abstract

**Background:**

Women's perceived control over condom use has been found to be an important determinant of actual condom use in some studies. However, many existing analyses used cross-sectional data and little quantitative information exists to characterize the relationships between perceived control and actual condom use among sex worker populations.

**Methods:**

We assessed the association between measures of perceived condom use control and self-reported use of male condoms employing data from a longitudinal pilot study among 192 sex workers in Madagascar.

**Results:**

In multivariable models, a lack of perceived control over condom use with a main partner and having a main partner ever refuse to use a condom when asked were both associated with an increased number of sex acts unprotected by condoms in the past week with a main partner (RR 1.86; 95% CI 1.21-2.85; RR 1.34; 95% CI 1.03-1.73, respectively). Conversely, no measure of condom use control was significantly associated with condom use with clients.

**Conclusion:**

Perceived control over condom use was an important determinant of condom use with main partners, but not clients, among sex workers in Madagascar. Programs working with sex workers should reach out to main and commercial partners of sex workers to increase male condom use.

## Background

Male condoms are one of the few effective methods currently available for the prevention of HIV/STI infection [1-3]. However, many women report having limited control over the use of male condoms with their sex partners [4-6]. Women reporting lower decision making power within relationships may be less likely to use condoms than women with greater power [6,7]. A meta-analysis of social power and normative support on condom use found that perceptions of condom use control generally had stronger associations among members of societal groups with less power, including females, younger individuals, ethnic-minorities and those with less education[8]. To date, there is limited quantitative information available on the relationship between perceived control over condom use and actual condom use patterns among sex workers from resource-constrained countries. Further, there is limited information examining these relationships by partner type (for example, with main partners *vs*. clients) or longitudinally. Research conducted among sex workers in resource limited settings has found that individual attitudes and beliefs have less influence on condom use than social and structural level factors[9-12]. Research among sex workers in Tanzania found that when sex workers decided to use condoms on their own or jointly with partners or clients, they were significantly more likely to use condoms than when the partner or client made the decision[13] Among sex workers in India, a number of different types of power were associated with consistent condom use, including control over the type of sex with clients, amount charged and economic independence[9].

We aimed to assess the association between various measures of condom use control and use of male condoms. We used data from a longitudinal pilot study that assessed the acceptability and feasibility of conducting a randomized trial to evaluate the effectiveness of the cervical diaphragm plus candidate microbicide or placebo gel in preventing infection with *C. trachomatis *(CT) and *N. gonorrhoeae *(GC) among sex workers in Madagascar

### Objectives

To describe baseline indicators of condom use control by type of sex partner and to assess whether poor condom use control is predictive of not using male condoms among female sex workers in Madagascar.

## Methods

We used data from a four-arm, partially-masked, randomized, prospective pilot study to assess the acceptability of evaluating the diaphragm plus candidate microbicide or placebo gel in preventing chlamydial and gonococcal infections among sex workers in four cities in Madagascar (Antananarivo, Antsiranana, Mahajanga, and Toamasina). Details of the pilot study are described in detail elsewhere [14]. Briefly, women were recruited as they were seeking care for STIs at public clinics in the four communities and through a community-based outreach program staffed by peer counselors. Eligibility criteria included having more than three different sex partners in the past month, less than 100% condom use in the past two weeks, ages 15-55 years, intending to stay in the area for the next month, not being pregnant or planning a pregnancy in the next two months, no known allergies to latex and no physical abnormality that precluded diaphragm use, and being able to give informed consent. Although self-reported sex work was not an inclusion criteria for the study, when asked if they had ever had sex to earn money, all women in this study responded "yes". The study was conducted between September and November 2005.

A total of 192 eligible women were enrolled. At enrollment all women were interviewed using a questionnaire which collected information on demographic, reproductive and sexual behavior characteristics. Women were asked to return for four weekly follow-up visits.

All women gave written informed consent for screening and again for enrollment. The institutional review boards at the University of North Carolina at Chapel Hill and at the Centers for Disease Control and Prevention, as well as the Comité d'Ethique auprès du Ministère de la Santé et du Planning Familial in Madagascar approved the study.

### Measures

At each follow-up visit, women reported on the number of sex acts in the past week during which male condoms were not used. Participants answered questions about sexual behaviors separately by partner type, and consequently we also conducted our analyses separately by partner type: 1) husband or boyfriend and 2) other partners (not a husband/boyfriend). In this paper we refer to husband/boyfriends as "main partners" and other partners as "clients". Our main outcome for each analysis was the number of sex acts not protected by a male condom during the previous week.

The main exposures were perceived control over condom use, and condom use dynamics with the main partner and with clients. The questionnaire included six questions about condom use control that were asked separately for main partners and for clients. The measures were used as proxies for gender power with main partners and clients. These measures included: 1) whether she had suggested using a condom at the last sex act; 2) whether she had ever refused to have sex because he would not wear a condom; 3) whether he had ever become violent when asked to use a condom; 4) whether he had refused to use a condom (ever for main partner, the last time they had sex for client); 5) whether he had become angry or argued when condom use was suggested (ever for main partner, the last time they had sex for client); 6) her perceived control over condom use, with responses dichotomized as a lot or complete versus none or little

### Analysis

All analyses were conducted using STATA (Version 9.0, College Station, TX).

We used negative binomial regression models to assess the association between six different measures of perceived condom use control and the number of unprotected sex acts in the past week. Because we collected outcome data on the same cohort of women at four weekly time points, we used generalized estimating equations (GEE) [15] to account for clustering with robust variance estimators and an exchangeable correlation structure. Unadjusted and adjusted models were developed. For multivariable models, variables were retained in the final model based on a manual backward elimination procedure (10% change in effect estimate) and based on *a priori *hypotheses.

## Results

The mean age of women in the sample was 30.2 years (standard deviation (SD): 7.8 years). Most women had limited education: the mean number of years of schooling was 5.7 (SD: 3.0 years). Nearly half the participants (45.5%) reported never being married, 37.6% were divorced or separated, 10.1% were cohabiting with a partner, 1.6% were married, and 5.3% were widowed. The median weekly income of women was 22,000 Malagasy Ariary (approximately USD 10.40) [Inter Quartile Range (IQR) 15,000-40,000 ma: USD $7.10-$19.00]. Women were asked whether their home had a list of ten items (tap water in house/property, hot running water, electricity, flush toilet, cellphone, TV, refrigerator, microwave, electric stove, car) the median number of items was 1 (range 0-6).

### Main partner characteristics at baseline

At baseline, less than half (41.8%, n = 79) of all women reported that they currently had a main partner. Of these, most (72.2%) described the partner as a boyfriend, 22.8% described him as a husband and the remaining percentage described him as a friend or "other". Of those with main partners, the mean length of the relationship was 2.6 years (SD: 2.9 years), and partner's mean age was 30.8 years (SD: 8.5 years). The majority of women with main partners reported that the partner was older: for 43.0% of women the partner was 5 or more years older; for 26.6%, the partner was 1-4 years older; and for 30.4%, the partner was the same age or younger than the participant. Women reported a mean of 2.3 sex acts (SD: 1.9 acts) per week with their main partner. The vast majority (70.8%) of women reported that their main partner probably or definitely had sex with other women in the past month. Alcohol use by the main partner during the last sex act was reported by 13.9% of participants, whereas 3.8% of women reported that they were under the influence of alcohol at the last sex act with their main partner.

### Client characteristics at baseline

At baseline, the median number of clients reported by women in the last week was 6 (IQR 4-9). More than half (54.2%) of women described their last client as "non-regular" (someone she had sex with only once or a few times) rather than "regular" (someone she had sex with on an on-going basis). Most women (68.2%) reported that they met their last partner on the street, with fewer reporting the woman's house (7.8%), "private place", nightclub, and hotel (approximately 4% for each). Twelve percent reported that their last client was under the influence of alcohol when they had sex while only 2% reported that they were under the influence of alcohol.

### Condom use at baseline

At baseline, condom use was more common with clients than with main partners (Table 1). More than 40% of participants reported never using a condom with their main partner, compared to 0.5% who never used male condoms with clients. Condom use at the last sex act with main partners was reported by 26.6% of women compared to 61.7% with clients.

**Table 1:** Measures of condom use and perceived condom use control among women by partner type at baseline among Malagasy sex workers, 2005.

	Main Partner	Client
	% (n = 79)	% (n = 189)
Frequency of condom use		
Never	44.3 (35)	0.5 (1)
Rarely	12.7 (10)	7.9 (15)
Sometimes	31.7 (25)	43.9 (83)
Almost always	10.1 (8)	40.7 (77)
Always	1.3 (1)	6.9 (13)
Condom use at last sex act	26.6 (21)	61.7 (116)
Woman suggested condom use with partner at last sex act	43.0 (34)	69.2 (130)
Woman ever refused to have sex with partner because he would not use a condom	51.4 (37)	79.7 (149)
Partner ever became violent when asked to use a condom	9.0 (7)	21.2 (40)
Perceived control over condom use		
None/little	52.6 (41)	40.2 (76)
A lot/complete	47.4 (37)	59.8 (113)
Partner refused to use a condom when asked*	65.8 (52)	32.3 (43)
Partner became angry or argued when condom use suggested*	44.3 (35)	13.5 (18)

*exact wording of the question and time period varied by partner type--see tables 2 & 3 for exact wording. Note that for main partners these questions were 'ever' while for clients it was the 'last client'

**Totals vary slightly due to missing data.

### Perceived control over condom use at baseline

At baseline, more women reported that they suggested condom use to their clients (69%) than to their main partners (43%) the last time they had sex. They also reported refusing to have sex more often if a client refused to use a condom, than if a main partner refused (Table 1). Just over 20% of women reported that a client had ever become violent when asked to use a condom, compared to 9% whose main partners had ever been violent when asked to use a condom. More than half of the women (52.6%) reported having no or little control over condom use with their main partners compared to 40.2% with clients.

### Sex acts unprotected by condoms over study follow-up

Summed over the full four-week follow-up period for all participants in the cohort, the number of sex acts where no condom was used was 49.2% (271/551 acts) with main partners and 35.6% (4257/11,958 acts) with clients.

### Associations between indicators of condom use control and acts unprotected by condoms Main partners

In unadjusted analyses, a lack of perceived control over condom use was associated with an increased likelihood of sex acts unprotected by male condoms with a main partner during the previous week (Table 2). This association remained significant in multivariable models adjusting for age, education, marital status, assets, study site, and study arm (Rate Ratio (RR) 1.86; 95% CI 1.21-2.85) (Table 2). Sex unprotected by male condoms was also more likely among women who reported that a main partner had ever refused to use a condom when asked (RR 1.34; 95% CI 1.03-1.73). Surprisingly, women who reported that a main partner had ever become angry or argued when asked to use a condom and who reported that a main partner had become violent when asked to use a condom were more likely to use condoms, but these associations were not statistically significant.

**Table 2:** Unadjusted and Adjusted Rate Ratios and 95% Confidence Intervals for associations between measures of perceived condom use control and the number of sex acts unprotected by a male condom with main partners in the past week among Malagasy sex workers, 2005.

Measure of perceived condom use control	Unadjusted RR (95% CI)	Adjusted RR** (95% CI)
Main partner ever refused to use a condom when asked		
No	1.0	1.0
Yes	1.51 (1.17-1.95)	1.34 (1.03-1.73)
Main partner ever became angry or argued when condom use suggested		
No	1.0	1.0
Yes	0.94 (0.74-1.19)	0.81 (0.62-1.05)
Amount of control over the use of condoms during sex with main partner		
A lot of control	1.0	1.0
Little or no control	1.41 (1.07-1.87)	1.86 (1.21-2.85)
Ever refused to have sex with main partner because he would not use a condom		
Yes	1.0	1.0
No	1.05 (0.83-1.32)	1.03 (0.87-1.22)
Main partner ever became violent when asked to use a condom		
No	1.0	1.0
Yes	0.84 (0.55-1.30)	0.85 (0.55-1.34)
Woman suggested condom use with main partner at last sex		
No	1.0	1.0
Yes	1.01 (0.80-1.27)	0.99 (0.84-1.16)

**Each perceived condom control variable was in a separate model adjusted for age, education, marital status, assets, study site, study arm

### Clients

With regard to unprotected condom acts with clients, in unadjusted models, women who reported little or no control over the use of condoms with clients were slightly more likely to report unprotected sex acts (RR 1.07; 95% CI 1.03-1.11). Women who reported never refusing sex with clients who would not wear condoms were slightly more likely to report acts unprotected by condoms with clients (RR 1.05; 95% CI 1.02-1.09). These effects did not remain significant in multivariable models, however (Table 3). In addition, women reported significantly fewer unprotected acts with non-regular client compared to regular clients (data not shown).

**Table 3:** Unadjusted and Adjusted Rate Ratios and 95% Confidence Intervals for associations between measures of perceived condom use control and the number of sex acts unprotected by a male condom with clients during the past week among Malagasy sex workers, 2005.

Measure of perceived condom use control	Unadjusted RR	Adjusted RR*
Client ever refused to use a condom when asked		
No	1.0	1.0
Yes	1.09 (0.81-1.46)	0.92 (0.73-1.17)
Client ever became angry or argued when condom use suggested		
No	1.0	1.0
Yes	0.98 (0.92-1.04)	0.99 (0.94-1.03)
Amount of control over the use of condoms during sex with clients		
A lot	1.0	1.0
A little/no control	1.07 (1.03-1.11)	1.03 (0.99-1.06)
Ever refused to have sex with client because he would not wear a condom		
Yes	1.0	1.0
No	1.05 (1.02-1.09)	1.03 (1.00-1.07)
Client ever became violent when asked to use a condom		
No	1.0	1.0
Yes	1.00 (.96-1.04)	0.98 (0.95-1.02)
Woman suggested condom use with client at last sex		
No	1.0	1.0
Yes	0.99 (0.95-1.03)	1.00 (0.97-1.03)

*Each perceived condom control variable was in a separate model adjusted for age, education, marital status, assets, study site, study arm and type of client

## Discussion

We found important differences in reported condom use between women's main partners and their clients. Measures of gender-based power, particularly perceived control over condom use, were associated with experiences of unprotected sex among main partners, whereas the effects were more attenuated and non-significant with clients.

Sex work in Madagascar is heterogeneous, different types and social categories of sex work have been reported [16,17]. In addition, it is not formally institutionalized through brothels or pimps thus most women work for themselves[16,17]. The majority of women in this study recruited clients on the street, markets or in bars and operate in poor neighborhoods. Importantly, the experience in this study and from other research with sex workers in Madagascar is that the distinction between main partners/boyfriend, called "sipas" in Malagasy, and clients is often fluid[16]. Clients can become sipas and vice-versa which makes the differentiation between roles within these relationships and the "rules" regarding condom use blurred. As observed in this study, unprotected sex was more common with regular clients than non-regular clients which may support the hypothesis that some clients become regular partners over time leading to more intimate relationships where condom use may no longer be wanted or expected.

Less than half of women in this study reported having a main partner. Nevertheless, among these women, more than half reported never or rarely using condoms with their main partner (even though the vast majority (70.8%) reported that their partner had other sex partners). At baseline, fewer women reported suggesting condom use to their main partner or refusing to have sex with a main partner if he would not use a condom compared to with clients. Lack of condom use with regular partners has been well documented among sex workers and is attributed to a desire both for greater intimacy with regular partners and as a means to differentiate work life from personal relationships [11,18,19]. Not using condoms with main partners may also represent a lower perceived risk of disease posed by main partnerships [18]. Limited condom use within main partnerships is well documented in non-sex worker populations as well [20,21]. As mentioned above, we did find that condom use with clients varied by client type (regular or non-regular); women reported significantly fewer unprotected acts with non-regular clients than with regular clients. It should be noted though that while the overall proportion of acts where no condom was used was greater among main partners than with clients, the total number of condomless sex acts was substantially greater among clients because of women's greater number of sex acts with clients. Therefore, prevention programs should avoid focusing too narrowly on sex acts with main partners. Programs should aim to increase the proportion of sex acts covered, whether these acts are with main partners or clients.

We found that women who perceived themselves as having little control over condom use with their main partners at baseline were significantly more likely to have unprotected sex with their main partner; however, the association for clients was weaker and non-significant. It may be that perceptions of control are more strongly associated with main partners where individual choice may play a greater role in determining whether condoms are used or not [18,22]. Perceptions of individual control may not exert as great an influence on condom use with clients as with main partners, because condom use with clients may be dictated by factors outside of the woman's individual perceived control (for example, price, client type, number of other women competing for clients, venue, *etc*.)[9,11,12]. It is for this reason that much of the recent literature on risk factors for HIV among sex worker populations and interventions aiming to address this risk have focused on the importance of environmental-structural factors[9,11,23].

### Limitations

The present analysis has a number of limitations. First, measures of condom use control were only captured at baseline and not at each follow-up visit. To the degree that condom use control varies by partner (particularly clients), it may be that perceptions of control do not align with actual behaviors due to changes in partners over time. This is less likely to be a problem with analyses of behavior with main partners, because most women will have the same main partner throughout the short, four-week follow-up period. In addition, time frames for the condom use control questions varied between main partners and clients. Condom use control questions for clients were asked about the last sex act while for main partners the time frame was "ever". This was done to improve recall for clients as women may have had many clients and remembering some of these events across partners would be more complicated than focusing on the last client. Second, this population was composed of women who reported that they did not consistently use condoms at baseline. Therefore, the generalizability of the findings to women who are able to use condoms consistently but were not eligible for this study is limited. However, the findings are very relevant to populations similar to ours - women who have little control over the use of barrier methods with partners - for whom alternative methods of prevention are most needed. In addition, the measures used in this study only examine one element of gender power and were by no means a comprehensive measure of gender power in this population. Last, sexual behavior and condom use data were self-reported and may suffer from social-desirability bias.

## Conclusions

We found that perceived condom use and a main partners refusal to use condoms when asked were important determinants of reported condom use with main partners among sex workers in Madagascar. Importantly, we found that measures of condom use control were more strongly associated with condom use with main partners than with clients. These findings underscore the importance of main partners in determining the use of male condoms. Ongoing prevention efforts with sex workers should offer programs that teach women condom negotiation skills and consider intervening with male partners to change norms with regard to condom use.

## Competing interests

The authors declare that they have no competing interests.

## Authors' contributions

AP was involved in the design of the study and in the analysis, write up and interpretation of the study findings. ANT and MK were involved in the design of the study and in the write up and interpretation of the study findings. MR, BR and KVD were involved in the design of the study, study implementation and data collection and in interpretation of study findings. All authors read and approved the final manuscript.

## Pre-publication history

The pre-publication history for this paper can be accessed here:

http://www.biomedcentral.com/1472-6874/10/4/prepub
